# Characterization and Modeling of Thermostable GH50 Agarases from *Microbulbifer elongatus* PORT2

**DOI:** 10.1007/s10126-021-10065-0

**Published:** 2021-09-30

**Authors:** Santi Rukminita Anggraeni, Marion B. Ansorge-Schumacher

**Affiliations:** 1 Professur Für Molekulare Biotechnologie, Technische Universität Dresden, Dresden, 01062 Germany; 2 Department of Marine Science, Faculty of Fisheries and Marine Science, Universitas Padjadjaran, Bandung, 45363 Indonesia

**Keywords:** *Microbulbifer elongatus*, GH50 agarase, β-Agarase, Thermostability, Natural agar

## Abstract

Viewing the considerable potential of marine agar as a source for the sustainable production of energy as well as nature-derived pharmaceutics, this work investigated the catalytic activity of three novel GH50 agarases from the mesophilic marine bacterium *Microbulbifer elongatus* PORT2 isolated from Indonesian coastal seawaters. The GH50 agarases AgaA50, AgaB50, and AgaC50 were identified through genome analysis; the corresponding genes were cloned and expressed in *Escherichia coli* BL21 (DE3). All recombinant agarases hydrolyzed β-*p*-nitrophenyl galactopyranoside, indicating β-glycosidase characteristics. AgaA50 and AgaB50 were able to cleave diverse natural agar species derived from Indonesian agarophytes, indicating a promising tolerance of these enzymes for substrate modifications. All three GH50 agarases degraded agarose, albeit with remarkable diversity in their catalytic activity and mode of action. AgaA50 and AgaC50 exerted exolytic activity releasing differently sized neoagarobioses, while AgaB50 showed additional endolytic activity in dependence on the substrate size. Surprisingly, AgaA50 and AgaB50 revealed considerable thermostability, retaining over 75% activity after 1-h incubation at 50 °C. Considering the thermal properties of agar, this makes these enzymes promising candidates for industrial processing.

**Supplementary Information:**

The online version contains supplementary material available at 10.1007/s10126-021-10065-0.

## Introduction

Agar is a sulfated polysaccharide produced by red agarophytes from the families *Gracilariaceae*, *Gelidiaceae*, *Pterocladiceae*, *Gelidiellaceae*, and *Ahnfeltiaceae*. The typical structure consists of repeated 3,6-α-anhydro-l-galactose and d-galactose linked by α-1,3 and β-1,4 glycosidic bonds (Usov 2011). Side chains such as methyl groups, sulfate esters, or pyruvate create a vast molecular diversity that considerably affects physical and chemical properties such as polarity, solubility, and gelling (Guiseley 1970; Lahaye and Rochas 1991). Overall, agar has unique gelling and stabilizing properties and has been used widely as supporting material for industrial and medical applications. Nowadays, it is also proposed as a potential biomass resource to replace petroleum feedstock (Park et al. 2020). Moreover, various potential biological activities of agar-derived sugars have been elucidated recently including anti-oxidant (Kobayashi et al. 1997), anti-diabetic, anti-obesity (Hong et al. 2017), anti-tumor (Lee et al. 2017), and immunomodulatory (Kang et al. 2017) effects making them interesting targets for pharmaceutics and cosmetics. For obtention of such sugars, enzyme-catalyzed degradation of polymeric agar is desirable since the released products are highly homogeneous, and the process is more environmentally friendly than chemical alternatives (Chi et al. 2012).

Agarases are glycoside hydrolases that cleave agar into smaller saccharides through a double- or single-displacement mechanism resulting in the retention or inversion of the anomeric carbon configuration (Lombard et al. 2014). The majority of agarases are produced by marine bacteria with a few exceptions originating from soil or symbiontic gut bacteria (Temuujin et al. 2011; Hehemann et al. 2010). Based on sequence similarity, the enzymes are classified into six families. Families GH16-16, GH50, and GH86 all include agarases that hydrolyze the β-1,4 glycosidic bonds in the agar molecules with a retaining mechanism, while agarases from the GH118 family cleave the same bond with an inverting mechanism. Families GH96 and GH117 contain inverting agarases cleaving the α-1,3 glycosidic linkages (Lombard et al. 2014).

Among the families, GH50 agarases show the most interesting variety in their catalytic mode while at the same time releasing a single product size, namely neoagarobiose (NA2) or neoagarotetraose (NA4). Most GH50 agarases are exo-agarases that perform bond cleavage from the chain ends of the substrate. Examples are Aga50D from *Saccharophagus degradans* 2–40 and AgaWH50A from *Agarivorans gilvus* WHO801 (Kim et al. 2010; Liang et al. 2017). A few members, such as DagB from *Streptomyces coelicolor* A3(2), show endo-exo hydrolytic action (Temuujin et al. 2011).

Considering the thermal properties of agar, enzymatic saccharification for industrial application requires agarases with maximum activity at high temperatures or with high thermostability (Park et al. 2020). Various such agarases have been isolated and characterized. However, most are GH16 agarases producing mixtures of differently sized neoagarooligosaccharides (Park et al. 2020). Among the GH50 agarases, only three thermostable enzymes have been described so far, namely AgaW and AgaB-4 from the soil bacteria *Cohnella* sp. LGH and *Paenibacillus agarexedens*, respectively (Li et al. 2015; Chen et al. 2018), and AgaL4 from *Microbulbifer pacificus* LD25, a bacterium isolated from a saltwater hot spring (Chen et al. 2019). All these thermostable GH50 agarases are metal-dependent and release NA2, NA4, and a mixture of NA4 and NA2, respectively, from agarose.

Here, we report the characterization of three novel GH50 agarases from the mesophile marine bacterium *Microbulbifer elongatus* PORT2. The organism was isolated from the Indonesian tropical coastal seawater and able to liquify agar plate medium and to utilize agar as a sole carbon source. Nevertheless, two of its GH50 agarases are distinctly thermostable. The number of GH50 agarases in *M. elongatus* PORT2 indicates a duplication event leading to similar protein folds, but different substrate specificity. We investigated the activities of the three GH50 agarases from *M. elongatus* PORT2 on natural agar extracted from Indonesian agarophytes and on standard commercial agarose. The degradation products of natural agar showed structural modification different from products derived from standard agarose. To the best of our knowledge, this is the first time that the degradation pattern and different activities of GH50 agarases from the same species have been compared and investigated using agarose as well as natural agar as substrates.

## Material and Methods

### Bacterial Isolation

*M. elongatus* PORT2 was isolated from the surface seawater of the coastal area Batu Karas, Pangandaran, West Java, Indonesia (7° 45′ 0″ S, 108° 30′ 0″ E). Seawater samples were taken with a sterile bottle, transported in a cold box, and processed for isolation using serial dilution on sterile KNO_3_ agar medium (yeast extract 1 g, KNO_3_ 0.2 g, agar 15 g, seawater 1 L, pH 8 ± 0.2). The colonies that formed a pit on the surface of the medium were further purified using the streak plate method. Incubation was performed for 24 to 48 h at 28 to 30 °C.

### Agar Extraction

Natural macroalgae, *Ulva* sp., *Gracilaria* sp., and *Gelidium* sp., were harvested from Sayang Heulang Beach, Pamengpeuk, Garut, West Java, Indonesia, in January 2018. The algae were cleaned from sands and marine biota, air-dried for 2 days, washed thrice with fresh water, and then sun-dried. For agar extraction, 5 g of dried algae was washed successively with distilled water, 700 mL distilled water was then added, and the sample was incubated in a water bath at 95 °C for 6–8 h. Finally, it was autoclaved at 121 °C for 15 min. The agar solution was filtered using Whatman paper grade 1. Two volumes of technical ethanol (99.5%) were added to 350 mL of the filtrate in the cold room for 2 h. The filtrate was centrifuged at 8000 rpm for 30 min at 4 °C and concentrated using a rotary evaporator at 40 °C for collecting the precipitate. The extract was freeze-dried and stored at − 20 °C. The resulting extract was designated as an alcohol-insoluble residue–containing agar (AIR).

### Genome Extraction, Sequencing, and Annotation

*M. elongatus* PORT2 was cultured aerobically in 10 mL of marine broth overnight on a rotary shaker at 180 rpm and 30 °C. The genomic DNA (gDNA) was extracted using the Wizard Genomic DNA Purification Kit (Promega, USA) according to the manufacturer’s instruction. The integrity of the gDNA extract was ascertained by agarose gel electrophoresis (0.8% w/v) and quantified with a NanoDrop 1000 spectrophotometer (Thermo Scientific, USA).

A genomic sequencing library of *M. elongatus* PORT2 was constructed from 1 ng of gDNA with the Nextera XT DNA Sample Preparation Kit (Illumina) according to the manufacturer’s instruction. The quality of the library was analyzed for fragment sizes of around 300–700 bp on an Agilent 2000 Bioanalyzer with Agilent High Sensitivity DNA Kit (Agilent Technologies). Sequencing on a MiSeq sequencer (Illumina; 2 × 250 bp paired-end sequencing, v3 chemistry) was performed by the Genomics Service Unit of the LMU Biocenter (Martinsried, Germany), resulting in 2.9 Mio raw reads. Raw reads were trimmed for quality (> Q20) and adapter sequences. De novo assembly was performed using CLC Genomics Server 8.0 (Qiagen) with the following parameters: bubble size = 194, minimum contig length = 1000, word size = 21, perform scaffolding = Yes, auto-detect paired distances = Yes, mismatch cost = 2, insertion cost = 3, deletion cost = 3, length fraction = 0.5, similarity fraction = 0.8.

The draft genome was submitted to the MicroScope platform for functional annotation. Genome quality was assessed using integrated tools within the MicroScope platform, CheckM analysis (Vallenet et al. 2017). The translated coding sequence output for agarase genes and proteins was further analyzed using several online platforms such as BLASTp against PDB and non-redundant protein database (nr) for pairwise comparison (https://blast.ncbi.nlm.nih.gov/Blast.cgi), ProtParam analysis for calculation of protein physical and chemical properties (https://web.expasy.org/protparam/), and SignalP 5.0 for signal peptide prediction (http://www.cbs.dtu.dk/services/SignalP/). A phylogeny tree was constructed for putative protein sequences of GH50 PORT2 by using the p-distance neighbor-joining method with 1000 bootstraps provided by the MEGA X software (Kumar et al. 2018).

### Cloning, Expression, and Purification of Recombinant GH50 Agarases

GH50 genes were amplified from the *M. elongatus* PORT2 gDNA using Q5 Taq polymerase (NEB) and specific primers (Supplementary Information Table S1). The signal peptides were excluded to enhance intracellular heterologous protein expression. The PCR products were purified using the Qiaquick PCR Purification Kit (Qiagen) and cloned into pFO4 (courtesy of the Glycobiology Group of the Biology Station of Roscoff, France) using a standard ligation strategy with different combinations of *Xho*I-*Nsi*I-*Bam*HI-*Eco*RI. The resulting plasmids, pFO4-AgaA50, pFO4-AgaB50, and pFO4-AgaC50, were validated using *Hinc*II mapping. Afterwards, they were transformed into *Escherichia coli* BL21 (DE3) cells according to the manufacturer’s manual for overexpression. Briefly, the cells were grown in 250 mL auto-induction medium ZYP-5052 (Studier 2005) supplemented with ampicillin (100 µg/mL) on a rotary shaker at 220 rpm for 48 h at 20 °C.

Unless specified, protein purification was performed at 4 °C. *E. coli* cells were harvested by centrifugation at 8000 g for 15 min and resuspended in cold lysis buffer (20 mM Tris–HCl pH 8.0; 500 mM NaCl; 1 mM EDTA, 0.1% (v/v) Triton X-100; 5 mM MgCl_2_) with fresh addition of DNAseI 10 mg/µL. The cell suspension was disrupted by sonication (LabSonic M Sartorius; 100% amplitude, 3 cycles, 1 min/cycle) and centrifuged at 12.000 g for 1 h. The soluble fraction was collected and loaded onto a HisTrap FF crude 5-mL column (GE) according to the manufacturer’s instruction. The column was washed using buffer A (Hepes 50 mM pH 8, imidazole 10 mM, and 500 mM NaCl). The binding protein was eluted using buffer B (Hepes 50 mM pH 8, imidazole 250 mM, 500 mM NaCl). The protein was desalted and concentrated using centrifugal ultrafiltration (Amicon® Ultra-15, Merck). The quality of protein expression was checked by SDS-PAGE 10% (w/v) using Coomassie Brilliant Blue R-250 for gel staining. Protein concentration was determined using a NanoDrop spectrophotometer (ND-1000 NanoDrop).

### Enzyme Assays for AgaA50 and AgaC50

Enzymatic reactions of AgaA50 and AgaC50 were measured using the artificial substrate *para-*nitrophenyl β-d-galactopyranoside (β-*p*npg). Appropriate enzyme and substrate controls were used during the experiment. A diluted enzyme was used with 1 mM β-*p*npg in the case of AgaA50 and 0.1 mM in the case of AgaC50 in a total volume of 1 mL. The sample was preincubated without enzyme for 2 min at the reaction condition. Continuous measurements were performed in triplicate. The release of *p*-nitrophenol was monitored at 405 nm on a Cary 60 UV–Vis system (Agilent). The enzyme activity was measured from the increase of *p*-nitrophenol concentration and corrected for the non-enzymatic hydrolysis of β-*p*npg. The concentration of *p*-nitrophenol in the sample was calculated using the *p*-nitrophenol standard curve in which the absorption coefficient (*ε*) of *p*-nitrophenol was defined specifically for each assay condition.

### Enzyme Assay for AgaB50

A proper enzyme dilution was used to hydrolyze 0.2% w/v of agarose in a total volume of 400 µL with appropriate enzyme and substrate controls. The enzyme activity was deduced from the increase of the d-galactose concentration and reported from the slope value of the initial reaction rate (µM/min) at a defined condition. The reaction was stopped by adding the dinitrosalicylic acid (DNS) reagent (1% 3,5-dinitrosalicylic acid; 0.2% phenol; 1% NaOH; 20% potassium sodium tartrate tetrahydrate) (w/v) (Miller 1959). The ratio of the DNS reagent to sample was 1:1. The mixtures were incubated in a 96-well thermal cycler (Advanced Primus 96) at 98 °C for 10 min and 4 °C for at least 15 min and measured at 540-nm absorbance using a 96-multiwell microplate reader (Tecan Infinite 200). All measurements were performed in triplicate. Reducing sugar in the sample was calculated using the d-galactose standard curve. One unit of enzyme activity was defined as the amount of enzyme that produced 1 μmol of d-galactose per minute at the defined reaction condition.

### Enzyme Characterization

The enzymatic reaction was performed at 50 or 60 °C. The pH range of activity was determined at a buffer concentration of 50 mM using sodium acetate for pH 5.1, Hepes for pH 6–8.6, Tris–HCl for pH 9.1, and CAPS for pH 10. Temperature range and stability were measured at 20 to 80 °C. Temperature stability was determined by incubating the enzyme at a defined temperature for 1 h, cooling the sample on ice for 5 min, and then measuring catalytic activity. The effects of chemical additives, namely NaCl, CaCl_2_, MgCl_2_, FeCl_3_·6H_2_O, dithiothreitol (DTT), ethylenediaminetetraacetic acid (EDTA), sodium dodecyl sulfate (SDS), and glycerol, were measured separately at various concentrations. Kinetic parameters of AgaB50 were defined at agarose concentrations from 1 to 10 mg/mL. Kinetic parameters of AgaA50 and AgaC50 were determined with β-*p*npg at concentrations between 1 and 15 mM and 0.1 to 10 mM, respectively.

### Product and Substrate Specificity Analysis

Substrate specificity was examined at a concentration of 0.2% (w/v) in ultrapure water (Milli-Q water). Agarose (peq-lab), agar kobeI (Roth), amylose (Roth), kappa-carrageenan (Sigma, Germany), and alcohol-insoluble residues (AIR) of *Gracilaria* sp. (AIRG), *Gelidium* sp. (AIRS), and *Ulva* sp. (AIRU) were heated to 95 °C and maintained at 50 °C to keep them dissolved. Other substrates such as laminarin (Sigma, Germany), porphyran (Carbosynth, UK), neoagarooctaose (NA8), neoagarohexaose (NA6), neoagarotetraose (NA4), and neoagarobiose (NA2) (Qingdao BZ Oligo Biotech, China) were soluble in cold water. NA8, NA6, NA4, NA2, d-glucose, and d-galactose were used as standards.

The products were analyzed using double-ascending thin-layer chromatography on precoated TLC sheet 0.2 mm silica gel 60 (Macherey–Nagel) with the solvent system water:acetic acid:n-butanol (1:1:2; v/v). Spots were visualized by shortly dipping the plate in 10% (v/v) H_2_SO_4_ in ethanol absolute and then drying with hot air at 150 °C for 5–10 min. Further, product analysis was performed using a high-performance liquid chromatography-refractive index detector (HPLC-RID) with a REZEX-RSO column (Phenomenex) and isocratic mobile phase ultrapure water (Milli-Q water) at 75 °C, a flow rate of 0.3 mL/min with a sample volume injection of 20 µL. The EZChrom Elite software (Knauer, Germany) was used for data acquisition and processing. The retention times of neoagarooligosaccharides used as standards were 14.6 min (NA8), 16.9 min (NA6), 20.9 min (NA4), 28 min (NA2), and 33.7 min (d-galactose).

### Protein Homology Modeling

Template search and model buildup were performed with the SWISSmodel server. The model quality was inferred from GMQE and QMEAN values (Waterhouse et al. 2018). Structure visualization, analysis, and comparison between the model and template were performed using UCSF Chimera (Pettersen et al. 2004).

### Accession Numbers of Genes

The gene sequences of agaA50, agaB50, and agaC50 as well as the sequence of the 16S rRNA from *M. elongatus* PORT2 were deposited in the GenBank database (NCBI) under accession numbers MT682142, MH996638, MT682143, and MH622756, respectively.

## Results

### Putative GH50 Agarases from *M. elongatus* PORT2

**Fig. 1:**
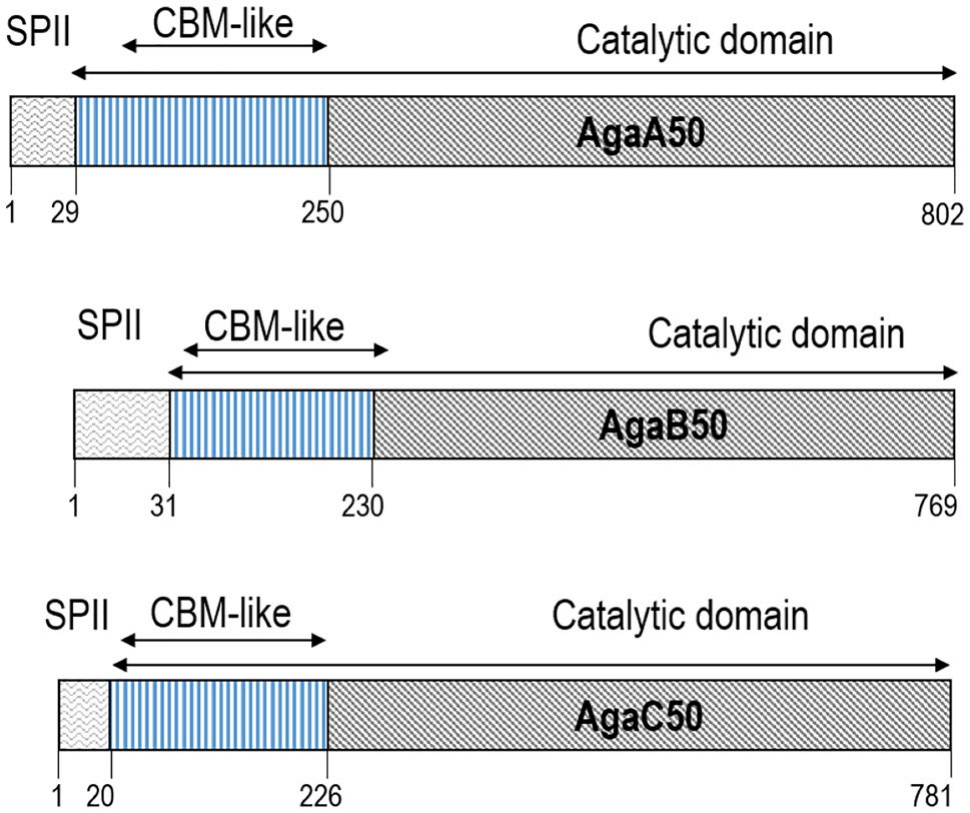
Modularity of GH50 agarases putative protein sequences in *M. elongatus* PORT2. Signal Peptide II (SPII) (gray wave); catalytic domain (black downward diagonal); carbohydrate-binding module-like (CBM-like) (vertical blue). Numbers below the bars represent positions of amino acids

Annotation and synteny analysis on the genome of *M. elongatus* PORT2 indicated three different putative GH50 agarases denoted as AgaA50, AgaB50, and AgaC50 (Fig. 1). The enzymes had different sequence lengths, calculated molecular weights, and pI. AgaA50 was encoded by a 2409-bp DNA and consisted of 802 amino acids with a total molecular weight of 89.9 kDa and a calculated pI of 4.8. AgaB50 included a 2310-bp DNA sequence encoding for 769 amino acids with a total molecular weight of 85.4 kDa and a calculated pI of 4.90. AgaC50 was encoded by a 2346-bp DNA and consisted of 781 amino acids with a total molecular weight of 87.7 kDa and a calculated pI of 4.99. Each of the putative GH50 agarases was fused to an N-terminal lipoprotein signal peptide (SPII) with cysteine at the + 2 position of the cleavage site indicating the enzymes as membrane-bound proteins (Gennity and Inouye 1991; Braun and Wu 1994; Seydel et al. 1999).

AgaA50, AgaB50, and AgaC50 shared 55.5%, 45.2,% and 52.4% sequence identity, respectively, with chain A of Aga50D, an exo-β agarase from *Saccharophagus degradans* 2–40 (SdAga50D) (Pluvinage et al. 2013). According to the Carbohydrate-Active enZYmes (CAZy) database, Aga50D is classified as a member of the GH50 family. Currently, members of this family are exclusively β-agarases consisting of pure exo β-agarases and endo-agarases with exo β-agarase capability. Interestingly, BLASTp analysis also revealed a homology of the C-terminus of *M. elongatus* PORT2 agarases to a conserved domain of GH42 β-galactosidase (pfam 02,449).

**Fig. 2:**
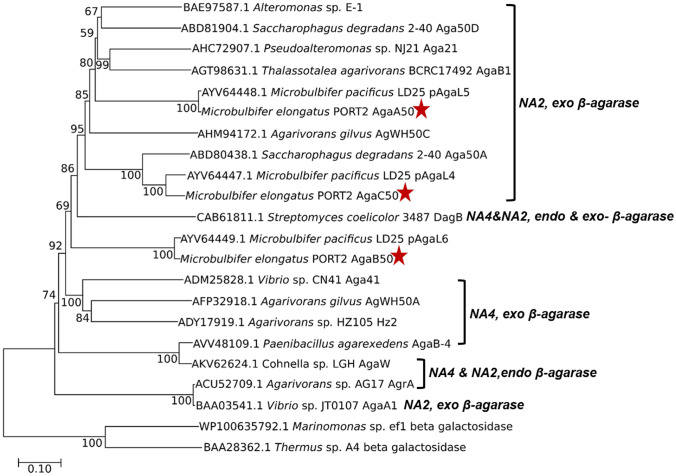
Neighbor-joining phylogeny tree showing the relationship of agarases from *M. elongatus* PORT2 (red stars) with known GH50 agarases including hydrolysis products and mode of action. The tree was constructed with the MEGA X software. The branch lengths indicate the evolutionary distances between enzymes using the p-distance method with 1000 bootstraps. Numbers on nodes denote the bootstrap values. Units indicate the number of amino acid differences per site

The relationship of the agarases from *M. elongatus* PORT 2 with other known GH50 agarases was visualized on a phylogeny tree. The tree showed bootstrap values ranging from 59 to 100%. AgaA50 and AgaC50 were clustered together within a solid clade of neoagarobiose-producing exo β-agarases (bootstrap value 95%), while AgaB50 was among neoagarotetraose and neoagarobiose producers (Fig. 2). The phylogeny relationships hinted at the mode of action and product spectrum of the enzymes.

AgaA50, AgaB50, and AgaC50 were overexpressed in *E. coli* BL21 (DE3) as N-terminal His_6_-tag proteins without the native signal peptides. The signal peptides were omitted to reduce incompatibility with heterologous protein expression and increase cytosolic expression. Precisely, 32, 26, and 22 amino acids were deleted from the N-terminus of AgaA50, AgaB50, and AgaC50, respectively. Purification from 250 mL batch cultures yielded 0.96 mg AgaA50, 5.1 mg AgaB50, and 13.8 mg AgaC50. The purified enzymes showed molecular sizes around 86, 82, and 85 kDa, respectively (Supplementary Information Fig. S1), corresponding to the expected N-terminally truncated proteins. Activity on the artificial substrates β-*p-*nitrophenyl galactopyranoside (β-*p*npg) indicated β-glycosidase characteristics.

### Action of Recombinant AgaA50, AgaB50, and AgaC50 on Natural Agar

**Fig. 3:**
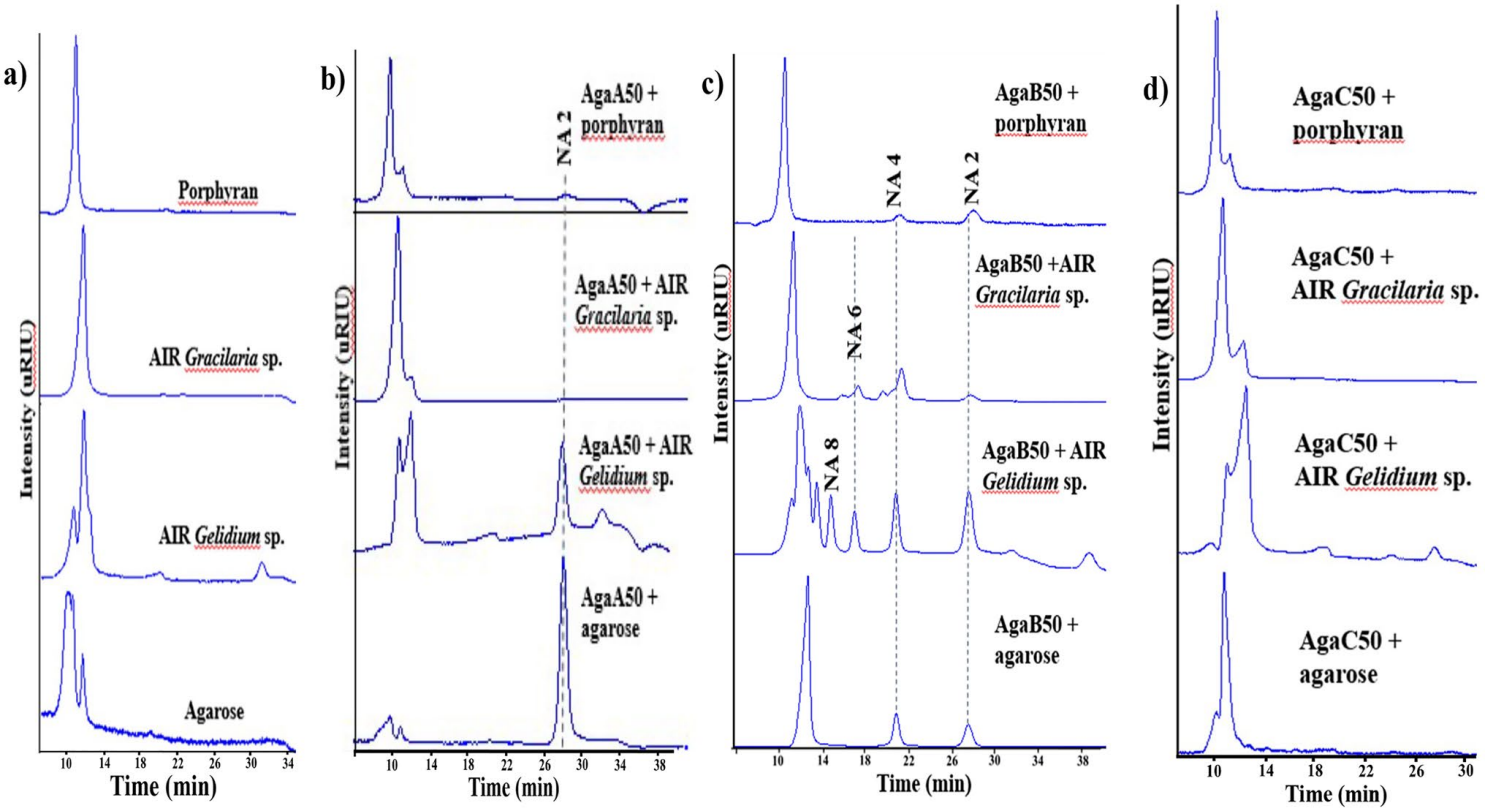
HPLC-RID analysis of *M. elongatus* PORT2 GH50 agarases acting on different agar polymers (0.2% w/v in ultrapure water). **a** Agar polymer peak profiles (AIR *Gelidium* sp. with a small glucose content); **b**–**d** Product profiles of **b** AgaA50, **c** AgaB50, and **d** AgaC50. Reaction conditions, 50 °C, 800 rpm, incubation overnight. Retention times of standard neoagarooctaose (NA8), neoagarohexaose (NA6), neoagarotetraose (NA4), and neoagarobiose (NA2) were 14.6, 16.9, 20.9, and 28 min, respectively

When incubated with natural polysaccharides from different sources, recombinant AgaA50 and AgaB50 showed preference for gelling agar such as agarose and AIR *Gelidium* sp. over viscous agar AIR *Gracilaria* sp. or water-soluble porphyran (Fig. 3). From AIR *Gelidium* sp. and agarose, AgaB50 released products similar to standard neoagarobiose (NA2) or neoagarotetraose (NA4). Larger neoagarooligosaccharides (NAOS) arose from AIR *Gelidium* sp. and AIR *Gracilaria* sp. The products from either AIR *Gracilaria* sp. or porphyran showed slightly longer retention times than NA4 and NA2 derived from agarose (Fig. 3b). AgaA50 released products with retention times similar to standard NA2 from AIR *Gelidium* sp. and agarose. Conversion of AIR *Gracilaria* sp. and porphyran was not observed with this enzyme (Fig. 3c). Notably, AgaC50 showed no activity on any of the tested macroalga polysaccharides (Fig. 3d). None of all three agarases was active on AIRU from *Ulva* sp., laminarin, amylose, or κ-carrageenan.

### Action Modes and Biochemical Properties of Recombinant AgaA50, AgaB50, and AgaC50

According to the findings on the degradation of various agar preparations by the GH50 agarases from *M. elongatus* PORT2, closer characterization revealed a remarkable diversity in the mode of action and biochemical properties of the three enzymes.

**Fig. 4:**
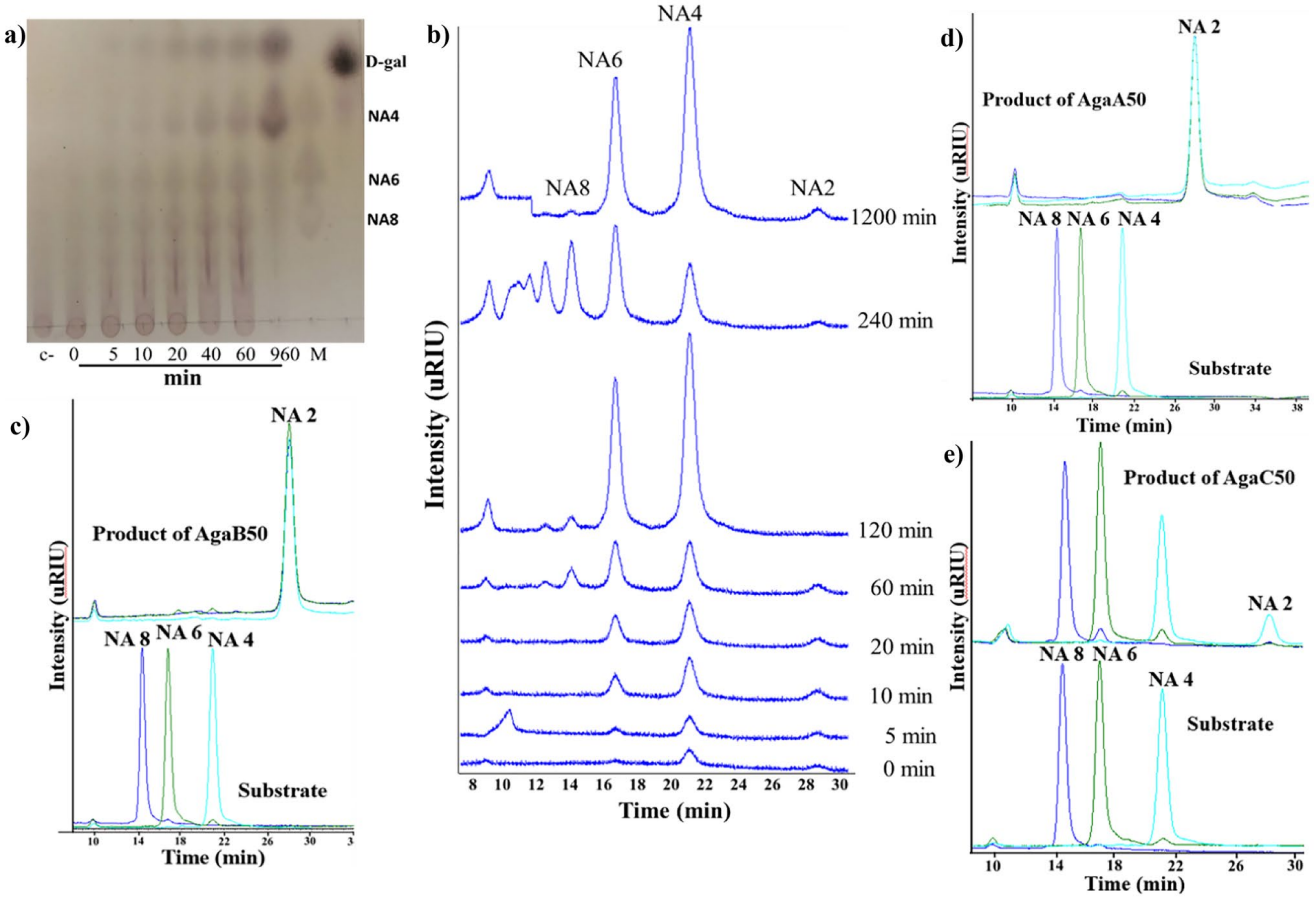
Substrate specificity of recombinant GH50 agarases. Time series reaction of AgaB50 on 0.2% (w/v) agarose in ultrapure water monitored by **a** TLC and **b** HPLC-RID. **c**–**e** HPLC-RID results from end point enzymatic reactions on neoagarooligosaccharides (NAOS) with **c** AgaB50, **d** AgaA50, and **e** AgaC50. All enzymatic reactions were carried out overnight at 50 °C and 800 rpm. The concentration of each NAOS was 0.5 mg/mL in ultrapure water. Retention times of neoagarooctaose (NA8), neoagarohexaose (NA6), neoagarotetraose (NA4), and neoagarobiose (NA2) were 14.6, 16.9, 20.9, and 28 min, respectively

**Fig. 5:**
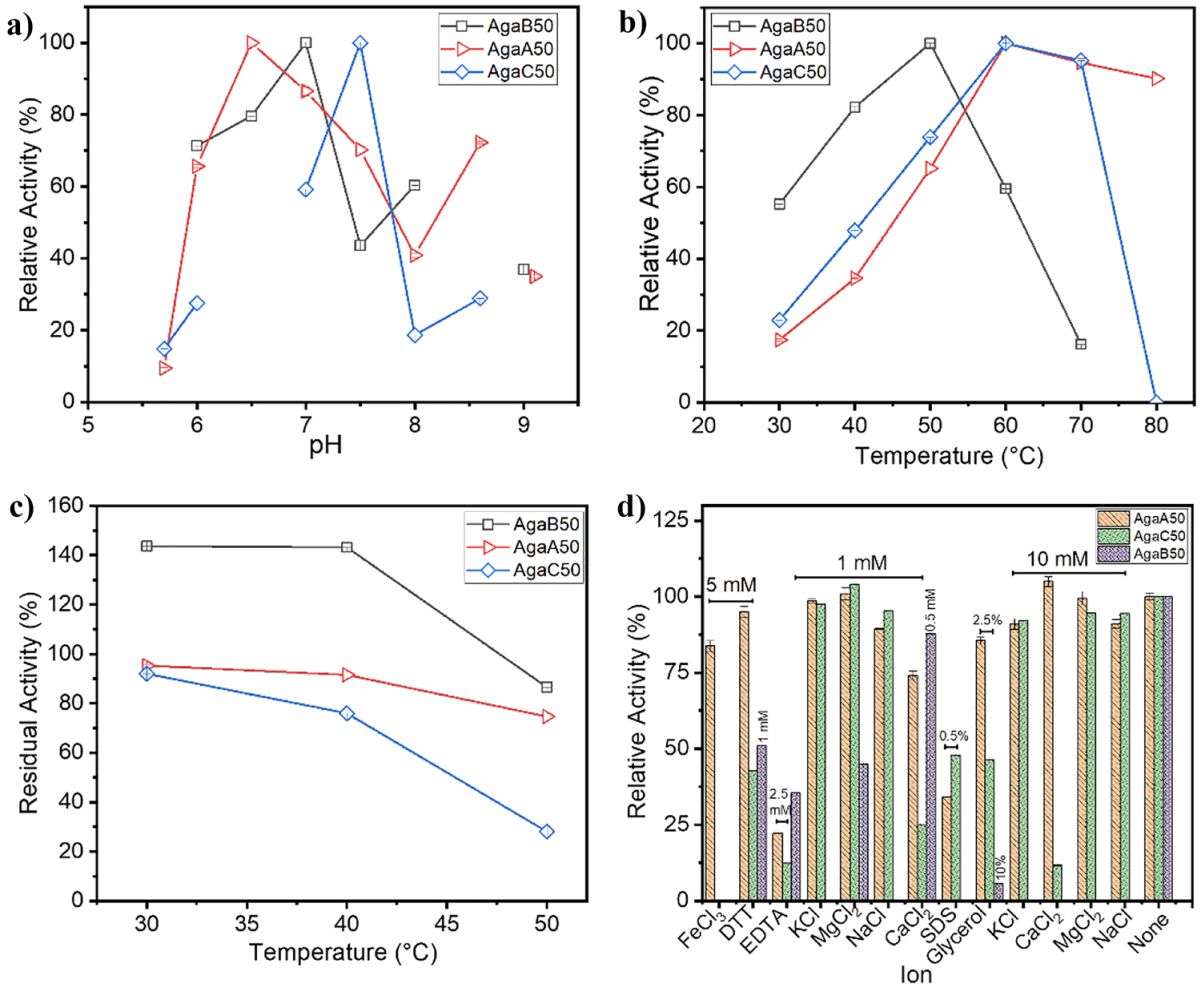
Catalytic activities of AgaA50, AgaB50, and AgaC50 in dependence on reaction conditions. **a** pH profiles at 50 °C; **b** temperature profiles at pH 7 (AgaA50 and AgaB50) and pH 7.5 (AgaC50), respectively; **c** activity after 1-h preincubation at elevated temperatures; **d** activity in the presence of additives. All data are the mean values from triplicate measurements. Error bars represent the deviation from the mean value. Agarose was used as substrate for AgaB50; β-pNPG was the substrate for AgaA50 and AgaC50. p-NPG para-nitrophenyl galactopyranoside, DTT dithiothreitol, EDTA ethylenediaminetetraacetic acid, SDS sodium dodecyl sulfate

AgaB50 degraded agarose gradually. TLC analysis detected neoagarotetraose (NA4) and neoagarobiose (NA2) as the major products. Neoagarooctaose (NA8) and neoagarohexaose (NA6) were also formed, but to a lesser extent (Fig. 4a). HPLC analysis revealed that the product spectrum changed after 4 h of reaction. While smaller NAOS were depleted, larger NAOS formed (Fig. 4b). This indicated AgaB50 as an endo β-agarase (Michel and Czjzek 2013). At the same time, AgaB50 was able to convert larger NAOS such as NA8, NA6, and NA4 yielding mostly NA2 (Fig. 4c). Thus, an additional exo β-agarase activity of the enzyme can be assumed (Michel and Czjzek 2013). AgaB50 demonstrated ability for agarose degradation in a wide pH and temperature range. It showed maximum activity of 242 U/mg ± 0.04 at 50 °C and pH 7 (Fig. 5a and b). However, *K*_M_ was rather high (Supplementary Information Fig. S2a), indicating only low substrate affinity towards agarose. Heat treatment at 20–40 °C for 1 h did not decrease the enzyme activity. After 1-h incubation at 50 °C, still 80% of the activity remained (Fig. 5c). However, the enzyme was strongly inhibited by all tested mono- and divalent cations (K^+^, Na^+^, Mg^2+^) as well as by the other tested additives such as dithiothreitol (DTT) or ethylenediaminetetraacetic acid (EDTA). The smallest effect was observed with Ca^2+^, in the presence of which AgaB50 maintained more than 75% activity. Glycerol and SDS turned out to be detrimental (Fig. 5d).

In contrast to AgaB50, AgaA50 and AgaC50 were not able to degrade agarose effectively under the assay conditions. The release of reducing sugars from agarose was below the detection limit of the DNS reagent (data not shown), but was qualitatively accounted for with HPLC-RID. AgaA50 released prominent amounts of NA2 from NA4, NA6, and NA8 (Fig. 4d). AgaC50 showed a more specific substrate preference by converting only NA4 into a small amount of NA2 (Fig. 4e). The HPLC profiles from NAOS hydrolysis displayed the formation of NA2 as the single product of both AgaA50 and AgaC50, suggesting exo-β-agarase activity. This resembled the activity of the exo-agarase AgaL4 from *Microbulbifer pacificus* LD25 (Chen et al. 2019) and was in agreement with the joint placement of the two enzymes in the phylogeny tree.

Since AgaA50 and AgaC50 were active on β-p-nitrophenyl galactopyranoside (β-pNPG), this artificial substrate was used instead of agarose to approximate the activity of both enzymes. AgaA50 displayed activity in a pH range between 6 and 9 with a maximum at pH 6.5 (Fig. 5a). The optimum temperature lie at 60 °C (Fig. 5b), and the enzyme maintained more than 70% of its activity after 1-h preincubation at 50 °C. After 1 h at 60 °C, it was fully inactivated (Fig. 5c). Na^+^, K^+^, Ca^2+^, Fe^3+^, glycerol, and DTT only slightly affected the enzyme activity. Negative effects were observed with SDS and EDTA. In contrast, the divalent cation Ca^2+^ slightly enhanced the activity of AgaA50 (Fig. 5d). The enzyme showed a Michaelis–Menten behavior on β-*p*npg with a *K*_M_ of 1.23 mM and a *v*_max_ of 0.028 mM/min (Supplementary Information Fig. S2b).

AgaC50 was also active in a wide pH range between 5.6 and 9.1 with a sharp maximum at pH 7.5 (Fig. 5a). Similar to AgaA50, the temperature optimum was at 60 °C (Fig. 5b). The enzyme retained more than 70% of its activity after 1-h preincubation at 40 °C, but after 1-h preincubation at a higher temperature, the activity was significantly decreased (Fig. 5c). A low concentration (1 mM) of the metal ions Na^+^, K^+^, and Ca^2+^ hardly affected the enzyme activity. With Mg^2+^ at a concentration of 1 mM, the activity was even enhanced. In contrast, Fe^3+^, EDTA, SDS, glycerol, and DTT significantly reduced AgaC50 activity. (Fig. 5d**)**. On β-*p*npg, AgaC50 also showed a Michaelis–Menten behavior with a *K*_M_ of 0.62 mM and a *v*_max_ of 0.95 µM/min (Supplementary Information Fig. S2c). Thus, it showed a stronger affinity to the substrate, but reached a considerably lower conversion rate than AgaA50.

The obtained results denote the recombinant enzymes AgaA50 and AgaB50 as the first thermostable GH50 agarases derived from a mesophilic marine organism. Similar to the thermostable GH50 agarases derived from thermophilic bacteria inhabiting unique niches such as AgaL4 from *Microbulbifer pacificus* LD25 (isolated from saltwater hot spring, activator Ca^2+^), AgaB-4 from *Paenibacillus agarexedens* (soil bacterium, activator Mn^2+^ and Co^2+^), and AgaW from *Cohnella* sp. LGH (soil bacterium, activator Mg^2+^, Ca^2+^, Na^+^, and K^+^) (Li et al. 2015; Chen et al. 2018, 2019), they maintained more than 75% of their activities after 1-h preincubation at 50 °C.

### Structural Modifications Within AgaA50, AgaB50, and AgaC50

Further insight into the divergent activity of the GH50 agarases from *M. elongatus* PORT 2 was derived from in silico studies of the enzyme structures. Homology models of all three enzymes were established based on the strong amino acid sequence similarity (> 40%) with chain A of well-characterized exo β-agarase Aga50D from *S. degradans* 2–40 (Kim et al. 2010; Pluvinage et al. 2013). The models considered a monomeric state and did not include the SPII signal sequences. The structure comparison between the three models and the template resulted in root mean square deviation (RMSD) values below 1 Å, indicating that SdAga50D PDB id 4BQ4.A was a suitable template (Fiser 2010; Jabeen et al. 2018).

**Fig. 6:**
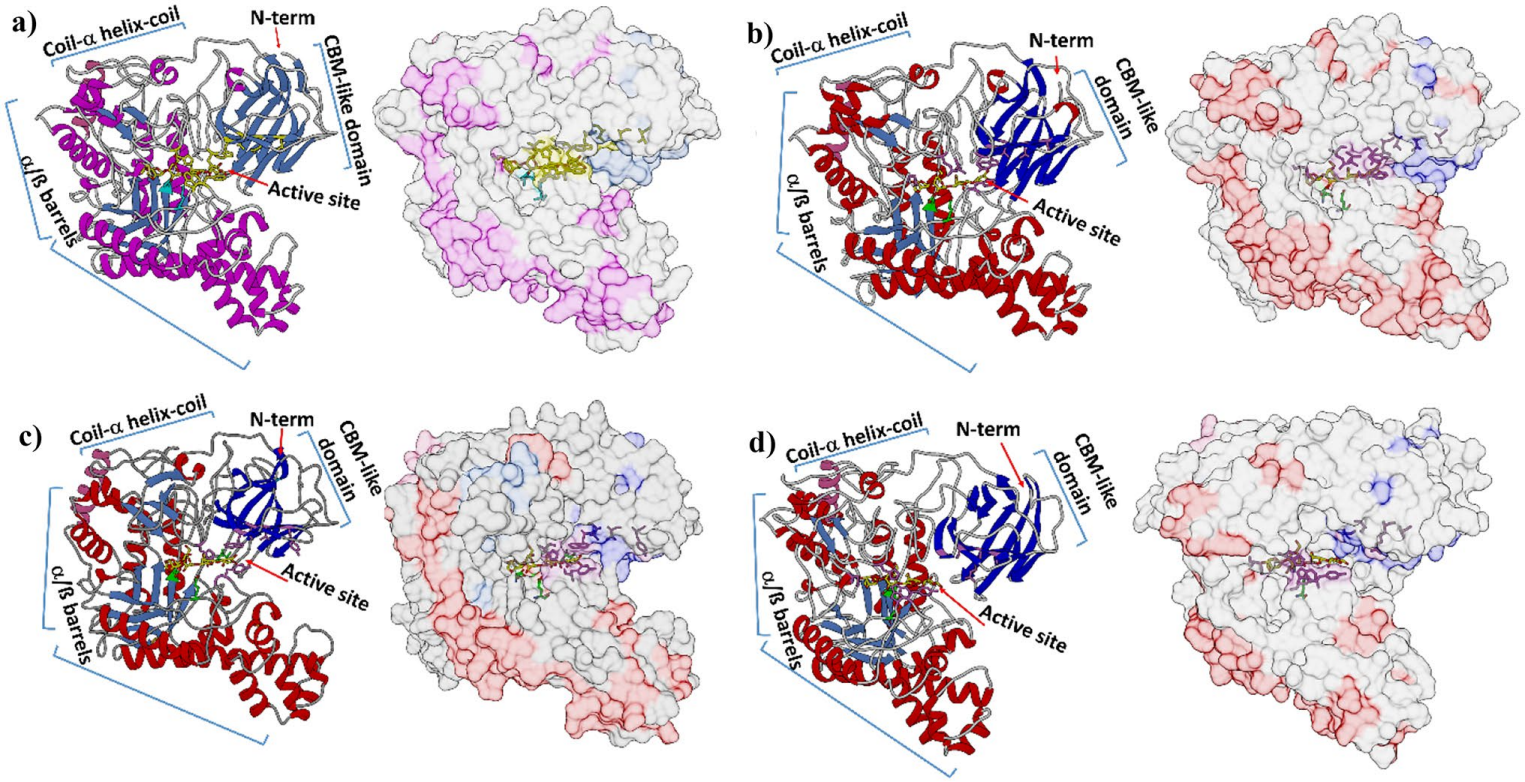
Homology structures of GH50 agarases from *S. degradans* 2–40 and *M. elongatus* PORT2, respectively, built by SWISSmodel on **a** SdAga50D (PDB id 4BQ4.A), **b** AgaA50, **c** AgaB50, and **d** AgaC50. N-terminal CBM-like domain (blue), coil-α-helix-coil (red), and C-terminal (α/β)_8_ barrels (red blue)

In general, the agarase structures consisted of a N-terminal carbohydrate-binding module-like (CBM-like) domain and a C-terminal (α/β)_8_ barrel domain linked via a coil-α-helix-coil (Fig. 6). In the active site, two glutamate residues about 5 to 5.4 Å apart were conserved indicating hydrolytic action in retaining mode (Davies and Henrissat 1995). Deletions and mutations in GH50 agarases from *M. elongatus* PORT2 compared to the Aga50D from *S. degradans* 2–40 are summarized in the Supplementary Information (Table S1).

**Fig. 7:**
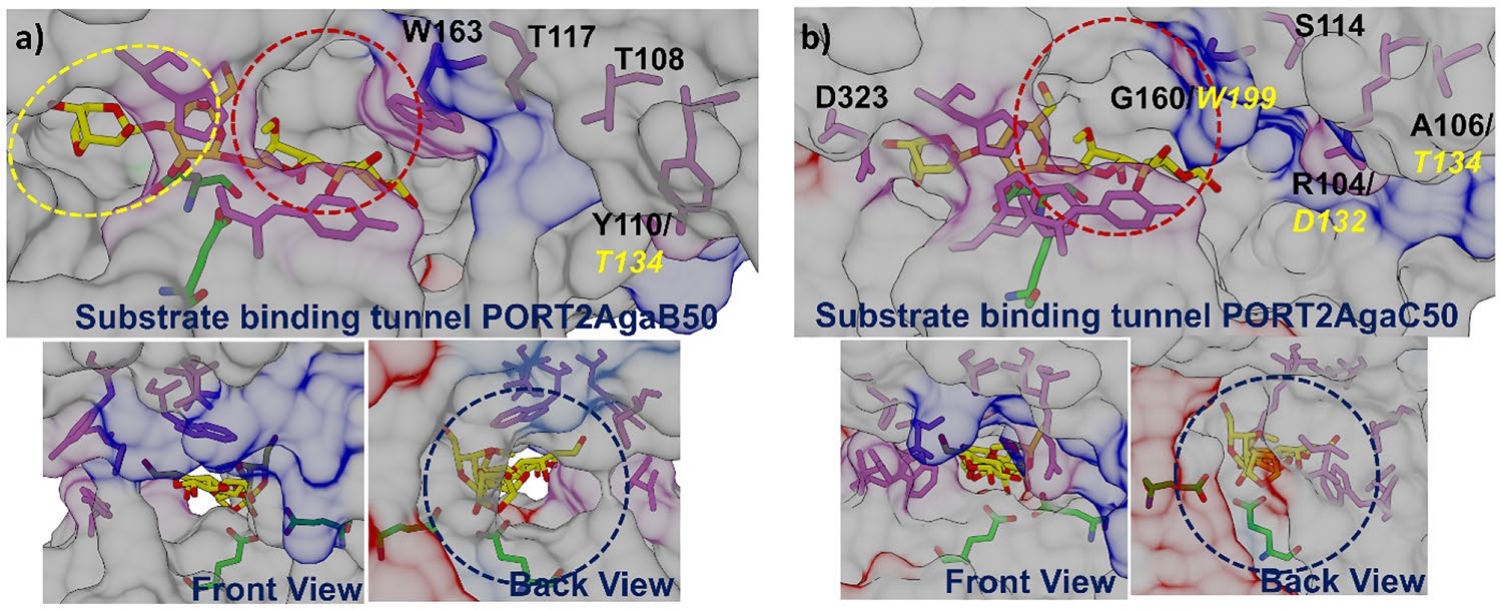
Modification of substrate-binding tunnels in GH50 agarases. **a** Opened tunnel end (yellow ellipse and blue-dashed cycle) and roof (red-dashed cycle) in AgaB50; **b** opened tunnel roof (red-dashed cycle) of AgaC50. Substrate-binding residues of AgaB50 or AgaC50 (orchid). Catalytic residues (green), neoagarotetraose (yellow–red sticks)

The closest structural resemblance to the template enzyme SdAgaD50 was found with AgaA50 complying with the overall similar activity of the two GH50 agarases. In both AgaA50 and AgaC50, the closed substrate-binding tunnel was conserved (Supplementary Information Fig. S3), which agrees with the distinct exo agarase activity of the two enzymes. In AgaC50, however, structural changes were found in the CBM-like domain. Most significant were the glycine residue at position 160 and the serine residue at position 114, which are both involved in substrate binding. Gly160 is homologous to Trp199 in SdAga50D, the + 2 subsite for the binding of 3,6-anhydro-l-galactose, while Ser114 is homologous to Thr142 in SdAga50D, responsible for the water-interposed formation of hydrogen bonds with 3,6-anhydro-l-galactose at + 3 and d-galactose at + 4 position (Fig. 7b). In addition, the substrate-binding tunnel in AgaC50 was shortened due to the missing of nine amino acids (Asp168–Asn176 in SdAga50D) from the roof formation (Fig. 7b). The changes might account for the observed disability of AgaC50 to recognize larger substrates such as NA6 and NA8 since accommodation and binding within the substrate-binding site would be hampered (Fig. 4e). They might also be connected with the feeble agarolytic activity of AgaC50. In another GH50 agarase with a deficiency for agarose cleavage, namely BpGH50 from *Bacteroides plebeius*, the N-terminal CBM-like domain and the first β-strand of the (α/β)_8_ barrel domain were found to be missing (Giles et al. 2017), indicating an important role of the CBM-like domain to assist and enhance enzyme–substrate vicinity and interaction in agarose conversion.

In the homology model of AgaB50, several structural changes were found that might vindicate the exo-endo mode of action of this enzyme in contrast to AgaA50 and AgaC50 (Fig. 6c). In particular, 26 amino acids corresponding to Val350–Ala375 in SdAga50D were deleted from the end block of the substrate-binding tunnel (Supplementary Information Fig. S3). Within this site, Asp362 was responsible for blocking the tunnel and forming hydrophobic interactions with 3,6-anhydro-α-l-galactose at the − 2 subsite (Pluvinage et al. 2013). Further twelve amino acids (Glu165–Asn176 in SdAga50D) were deleted in the CBM-like domain forming the tunnel roof (Fig. 7a). The modifications shorten the tunnel and create a more open topology. In the substrate-binding subsite, Tyr110 replaces the homologous Thr134 in SdAga50D, which is responsible for the water-mediated formation of a hydrogen bond with d-galactose at the + 5 subsite (Fig. 7a). The aromatic ring of the tyrosine residue possibly enables a stronger binding of longer oligosaccharides. Together, the deletions and mutations might augment the binding affinity of AgaB50 for longer and larger substrates and consequently shift the activity from a pure exo agarase to an exo-endo agarase. In addition, the hydrolysis/transglycosylation equilibrium is probably affected as indicated by the changes in NA4 and NA2 formation after prolonged reaction time (Fig. 4b).

## Discussion

Three homologous GH50 agarases with considerably varying catalytic activity and biochemical properties were identified in the marine bacterial isolate *M. elongatus* PORT2 offering potential for the processing of agar as a source for the sustainable production of energy as well as nature-derived pharmaceutics. In particular, they show hydrolytic activity not only on commercial agarose, but also on natural agar releasing NAOS derivatives that might have novel biological activities.

The broad acceptance of diverse natural agars by AgaA50 and AgaB50 along with the observed slight variations in product sizes indicated a promising tolerance of the enzymes for substrate modifications and the concomitant ability of *M. elongatus* PORT2 to survive under varying substrate conditions. Long since it has been described that the molecular characteristics of agar polysaccharides change with bio-ecological factors such as producing species, harvesting period, nutrient availability, and hydrodynamic conditions (Usov 2011; Lahaye and Rochas 1991; Rebello et al. 1997; Sousa et al. 2013), which then influence the physical properties, particularly the polarity, solubility, and gel formation of the agar (Guiseley 1970; Lahaye and Rochas 1991). This must also severely affect degradability and thus the nutritional value of the polymers as substrates.

The positive effects of the divalent cations Ca^2+^ and Mg^2+^ on the activities of AgaA50 and AgaC50, respectively, and the concurrent negative effect of EDTA indicate that these two GH50 agarases are metal-dependent (Klebe 2013; Pereira et al. 2017). This corresponds with the features of known thermostable GH50 agarases (Li et al. 2015; Chen et al. 2018, 2019). In contrast, AgaB50 was not only deactivated by EDTA, but was also negatively affected by any of the ions or other chemicals added to the reaction. Thus, a metal dependency of this enzyme cannot be deduced safely at this point. The effects of EDTA could also result from an overall sensibility of the enzyme structure for changes in its environment (Bisswanger 2014; Robinson 2015; Lopina 2017). The negative effect of the anionic detergent SDS on the activities of all three novel agarases from *M. elongatus* PORT2 indicated their localization in the cell membrane. The anionic detergent has been found to disrupt hydrophobic or protein–protein interactions of membrane-bound proteins or enzymes in the first place (Walker 2009). This is in agreement with the N-terminal lipoprotein signal peptide fused to the enzymes, which was deduced from genome analysis.

Since the *K*_M_ and *v*_max_ values of AgaA50 and AgaC50 were derived from the conversion of an artificial substrate while known GH50 agarases were characterized on natural substrates such as agar or neoagarooligosaccharides, their catalytic activity can hardly be compared to that of the GH50 agarases described so far. The *K*_M_ of AgaB50 on agarose indicates a comparably low affinity to this substrate among the known GH50 agarases. However, this complies with the observed endo-exo catalytic action of AgaB50, which relates this enzyme closer to DagB from *Streptomyces coelicolor* A3(2) (Temuujin et al. 2011) than to the known thermostable GH50 agarases, as also indicated by its placement in the phylogeny tree.

Most remarkably, two of the GH50 agarases revealed considerable thermostability albeit coming from a distinctly mesophilic organism. In fact, to our knowledge, they are the first thermostable GH50 agarases derived from a marine mesophilic bacterium so far. They are slightly inferior to the AgaL4 isolated from the thermophilic *M. pacificus* LD25 (Chen et al. 2019), but demonstrate the hardly tapped potential of mesophilic marine organisms as sources for enzymes with industrial relevance. They might even indicate *Microbulbifer* as a particularly promising genus with regard to agar processing.

A structural explanation for the observed thermostability of AgaA50 and AgaB50 cannot be offered at this time since diverse factors such as the number of hydrogen bonds, surficial proline and arginine residues, salt bridges, and structure compactness might be responsible (Sterner and Liebl 2001). A comparison of the GH50 agarases from *M. microbulbifer* PORT2, the structurally related mesophilic SdAga50D, and other thermostable GH50 agarases with regard to these features gave no significant differences (data not shown). However, shorter hairpin loops could be found in the thermophilic enzymes compared to the mesophilic SdAga50D, which might contribute to stability by increasing structure compactness.

The occurrence of paralogous GH50 agarases in *M. elongatus* PORT2 implies gene duplication that maintains a similar function with diverge specificity (Qian and Zhang 2008). Generally, this is a costly event since it may cause higher energy consumption and significantly reduce the organism’s fitness (Wagner 2005). However, the divergence in structure and functionality found in the GH50 agarases from *M. elongatus* PORT2 indicates evolution towards a larger substrate scope with an increase of the ecological fitness of the organism.

## Supplementary Information

Below is the link to the electronic supplementary material.

Supplementary file1 (DOCX 1339 KB)

Supplementary file2 (XLSX 10 KB)

## Declarations

Ethics approval is not applicable because the article does not contain any studies with human participants.

## Abbreviations

NAOS: Neoagarooligosaccharides
SDS: Sodium dodecyl sulfate
PAGE: Polyacrylamide gel electrophoresis
DNS: 3,5-Dinitrosalicylic acid
TLC: Thin-layer chromatography
EDTA: Ethylenediaminetetraacetic
