# Supplementary material for: Characterization and Modeling of Thermostable GH50 Agarases from Microbulbifer elongatus PORT2
Source: Mar Biotechnol (NY). 2021 Sep 30;23(5):809–20. doi: 10.1007/s10126-021-10065-0 (PMC8551122; doi:10.1007/s10126-021-10065-0)
Supplement: Supplementary file 1 — Supplementary file1 (DOCX 1339 KB) [file 10126_2021_10065_MOESM1_ESM.docx]

**Supplementary Information**

**
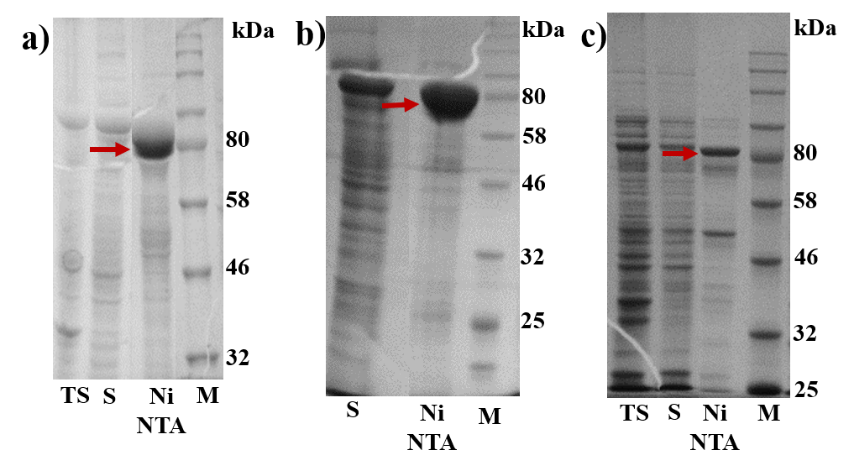
**

**Fig. S1** Recombinant GH50 agarases of *M. elongatus* PORT2 overexpressed in *E. coli* BL21 (DE3). The proteins were purified using Ni-NTA column, desalted and concentrated using centrifugal ultrafiltration (Amicon 15, Millipore Merck, USA), and the quality was detected on SDS PAGE (*red arrows*): a) AgaA50, b) AgaB50, c) AgaC50. Total protein (TS); Soluble protein (S); elution fraction from Ni-NTA IMAC (Ni-NTA); Color prestained protein marker, broad range (11-245 kDa) New England Biolabs (M).

**Table S1** Specific Primers for cloning of agarase genes

| Gene | size  (bp) | Restriction  Enzyme | Primer pairs | Cloning  Host | Plasmid &  Size (bp) |
| --- | --- | --- | --- | --- | --- |
| agaA50 | 2409 | BamHI/  EcoRI | F: 5'-ttttttggatccgagcagaaaggtggcgagactg-3'  R: 5'-aaaaaagaattctcactcggcaggcttcacatcg-3' | *E.coli* DH5a NEB | pME1-8002 |
| agaB50 | 2310 | XhoI-NsiI | F: 5'-ttttttctcgagctgctgtctgcctgtggtcagt-3'  R: 5'-aaaaaagaatttactctggcgccactgccaatt-3' | *E.coli* DH5a NEB | pME2-7930 |
| agaC50 | 2346 | XhoI-NsiI | F: 5'-ttttttctcgagaatgatgtccggtccacgattaca-3'  R: 5'-aaaaaaatgcatttactctggcgccactgccaatt-3' | *E.coli* DH5a NEB | pME3-7981 |


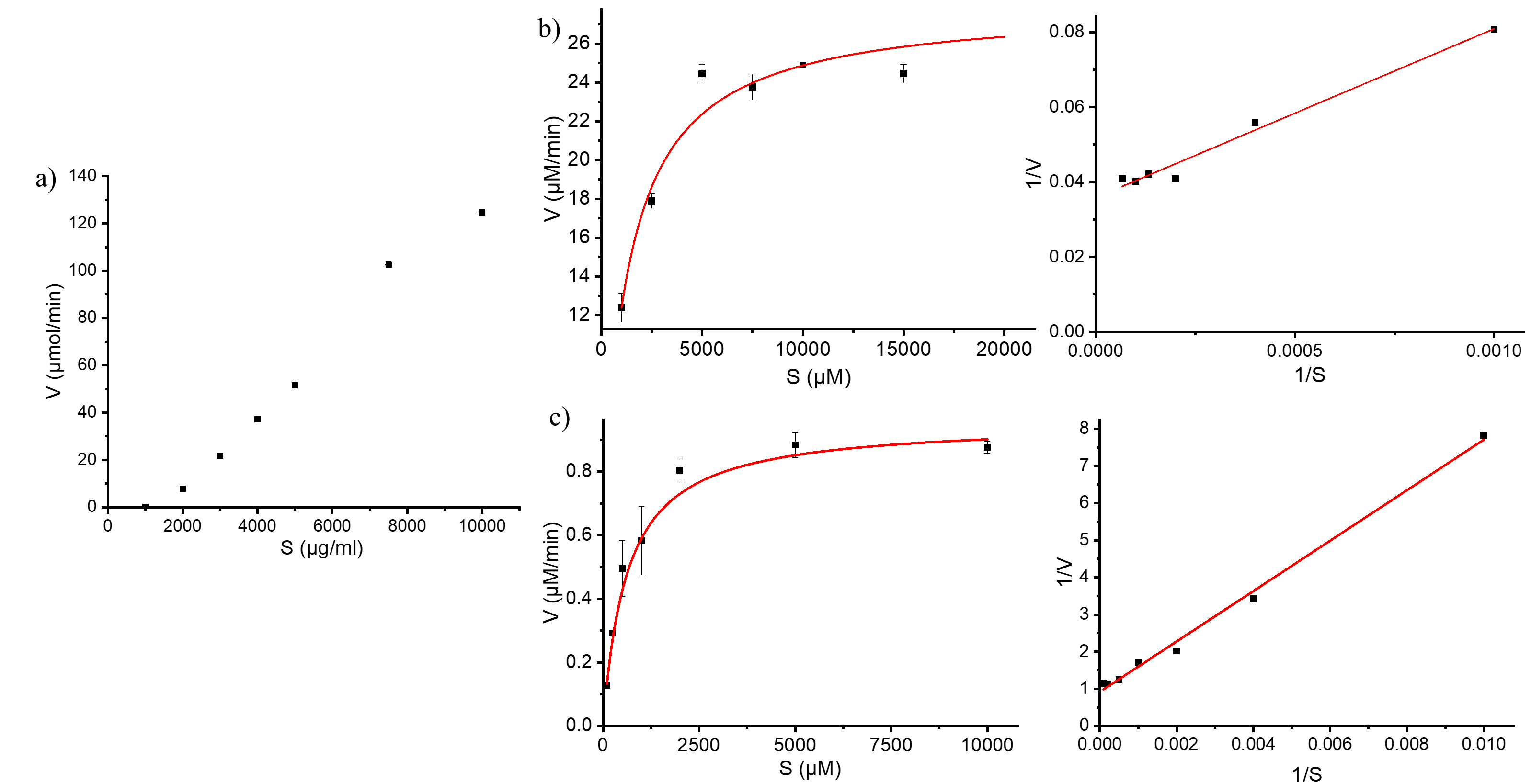


**Fig. S2** Effect of substrate concentration on the activity of recombinant GH50 agarases of *M. elongatus* PORT2: a) Activity of AgaB50 on various concentrations of agar in 50 mM of Hepes pH 7 at 50 °C; b) and c) AgaA50 and AgaC50 activities on various concentration of β-pNPG in 50 mM Hepes pH 6.5 and pH 7, respectively.


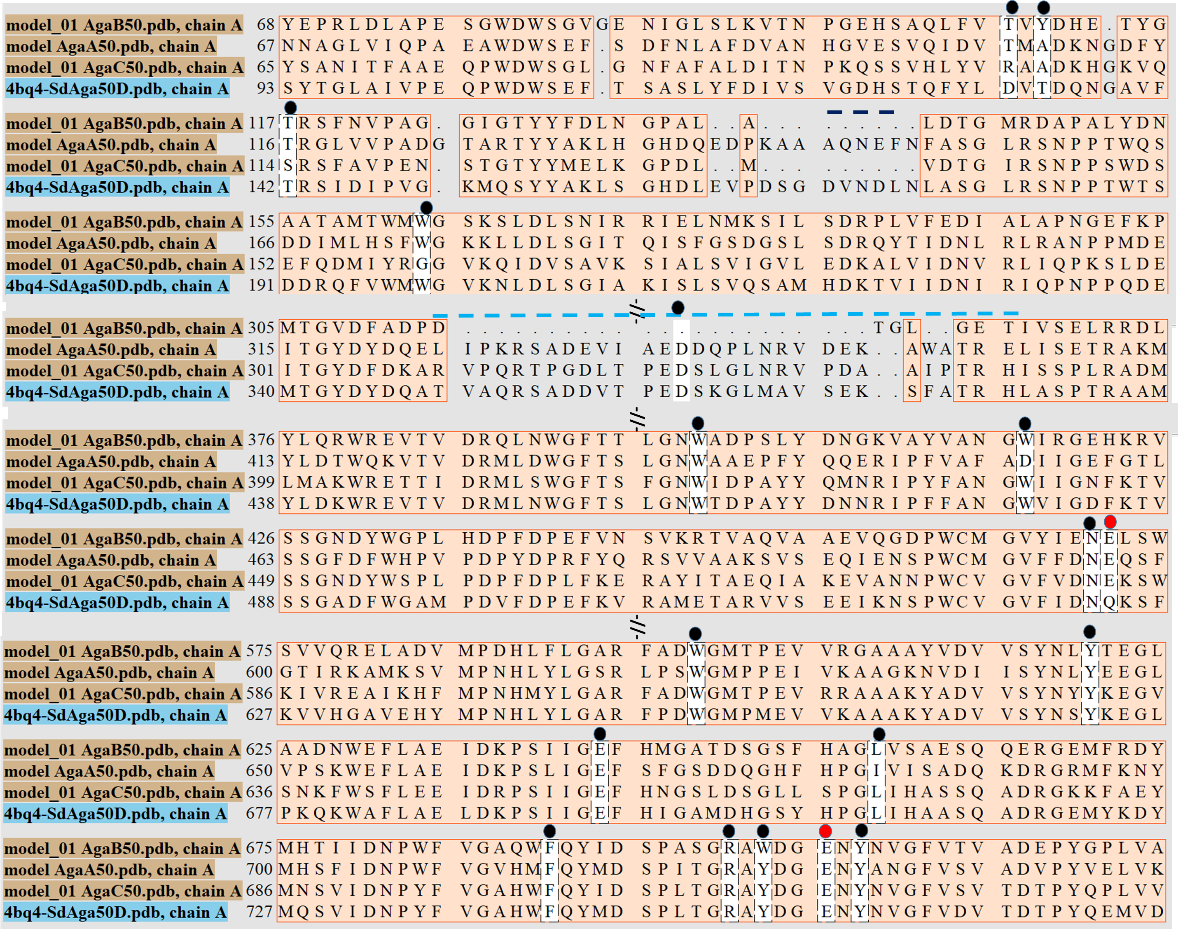


**Fig. S3** Multiple alignments between GH50s of PORT2 with the template SdAga50D; *black dots* are substrate-binding residues; *red dots* are catalytic residues; the *black-dashed line* is a modification at the CBM-like domain, the *blue-dashed line* is the end loop of the active site tunnel.
